# COVID-19 vaccination and allergen immunotherapy (AIT) – A position paper of the German Society for Applied Allergology (AeDA) and the German Society for Allergology and Clinical Immunology (DGAKI) 

**DOI:** 10.5414/ALX02245E

**Published:** 2021-08-24

**Authors:** Ludger Klimek, Oliver Pfaar, Eckard Hamelmann, Jörg Kleine-Tebbe, Christian Taube, Martin Wagenmann, Thomas Werfel, Randolf Brehler, Natalija Novak, Norbert Mülleneisen, Sven Becker, Margitta Worm

**Affiliations:** 1 Center for Rhinology and Allergology, Wiesbaden,; 2 Department of Otorhinolaryngology, Head and Neck Surgery, Section of Rhinology and Allergy, University Hospital Marburg, Philipps-Universität Marburg, Marburg,; 3 University Hospital for Pediatrics and Adolescent Medicine, Children’s Center Bethel, University of Bielefeld,; 4 Allergy Center Westend, Berlin,; 5 Department of Pneumology, University Hospital Essen – Ruhrlandklinik, Essen,; 6 Department of Otorhinolaryngology, University Hospital Düsseldorf, Düsseldorf,; 7 Department of Dermatology, Allergology and Venerology, Hanover Medical School,; 8 Department of Dermatology, University Hospital Münster, Division of Allergology, Occupational Dermatology and Environmental Medicine, Münster,; 9 Department of Dermatology and Allergy, Polyclinic for Dermatology and Allergology, Bonn,; 10 Asthma and Allergy Center, Leverkusen,; 11 Clinic for Otorhinolaryngology, University Hospital, Tübingen, and; 12 Allergology and Immunology, Clinic for Dermatotology, Venerology and Allergology, Charité Universitätsmedizin Berlin, Germany

**Keywords:** allergen immunotherapy, allergic rhinitis, asthma, COVID-19, SARS-CoV-2

## Abstract

Background: Vaccinations against severe acute respiratory syndrome coronavirus type 2 (SARS-CoV-2) are intended to induce an immune response to protect against infection/disease. Allergen immunotherapy (AIT) is thought to induce a (different) immune response, e.g., to induce tolerance to allergens. In this position paper we clarify how to use AIT in temporal relation to COVID-19 vaccination. Four SARS-CoV-2 vaccines are currently approved in the EU, and their possible immunological interactions with AIT are described together with practical recommendations for use. Materials and methods: Based on the internationally published literature, this position paper provides specific recommendations for the use of AIT in temporal relation to a SARS-CoV-2 vaccination. Results: AIT is used in 1) allergic rhinitis, 2) allergic bronchial asthma, 3) insect venom allergy, 4) food allergy (peanut). Conclusion: For the continuation of an ongoing AIT, we recommend an interval of 1 week before and after vaccination for subcutaneous immunotherapy (SCIT). For sublingual immunotherapy (SLIT) and oral immunotherapy (OIT), we recommend taking them up to the day before vaccination and a break of 2 – 7 days after vaccination. Initiation of a new SCIT, SLIT, or OIT should be delayed until 1 week after the day of the second vaccination. For SCIT, we generally recommend an interval of ~ 1 week to COVID-19 vaccination.

**German version published in Allergologie, Vol. 44, No. 5/2020, pp. 339-348.**

## Introduction 

### Allergen immunotherapy 

Allergen immunotherapy (AIT) is the only causally effective therapy for which long-term clinical benefit has been demonstrated in allergic respiratory diseases, for example, allergic bronchial asthma or allergic rhinoconjunctivitis and other allergic diseases [1]. Since its first description more than 100 years ago (1911) [2], AIT has been an established and internationally recognized procedure for the treatment of allergies. 

AIT induces immune tolerance to a specific, individually relevant allergen [3]. Systematic meta-analyses have confirmed that AIT significantly reduces symptoms of allergic disease and the amount of necessary antisymptomatic medication in patients with allergic asthma [4] and allergic rhinoconjunctivitis [5]. 

This is true for both subcutaneous immunotherapy (SCIT) [6, 7] and sublingual immunotherapy (SLIT) [8]. 

The risk of patients with allergic rhinitis developing asthma is reduced by AIT [9, 10]. AIT is also effective in patients with IgE-mediated food allergy [11, 12] and insect venom allergy [13]. In addition, the cost-saving effect of this disease-modifying therapeutic option [14, 15, 16] has been demonstrated. 

After the World Health Organization (WHO) declared the severe acute respiratory syndrome coronavirus type 2 (SARS-CoV-2)-transmitted corona virus 19 (COVID-19) infectious disease pandemic in March 2020 [17], numerous position papers and recommendations for action from international and national allergological societies on the management of allergological diseases and their therapies in the pandemic have been published [18, 19, 20, 21, 22, 23, 24, 25, 26, 58, 59, 60, 61, 62, 63]. 

For the two mRNA-based vaccines currently approved in Europe (Comirnaty from BioNTech [27]; MRNA-1273 from Moderna [28]) and two vector-based vaccines (CHAdOx1-S from AstraZeneca [29]; Ad26. COV2-S from Johnson & Johnson [30]), recommendations for allergological risk assessment of COVID-19 vaccinations have been published in cooperation of AeDA (Ärzteverband Deutscher Allergologen), DGAKI (Deutscher Gesellschaft für Allergologie und Klinische Immunologie), and GPA (Gesellschaft für Pädiatrische Allergologie und Umweltmedizin) [31, 32, 33]. 

Based on the technical and directions for use of the four approved COVID-19 vaccines, AIT is not a contraindication [27, 28, 29, 30]. 

On this basis, scientific societies have published recommendations for COVID-19 vaccinations for patients undergoing biologic therapy [25, 26, 64]. 

**Figure 1:**
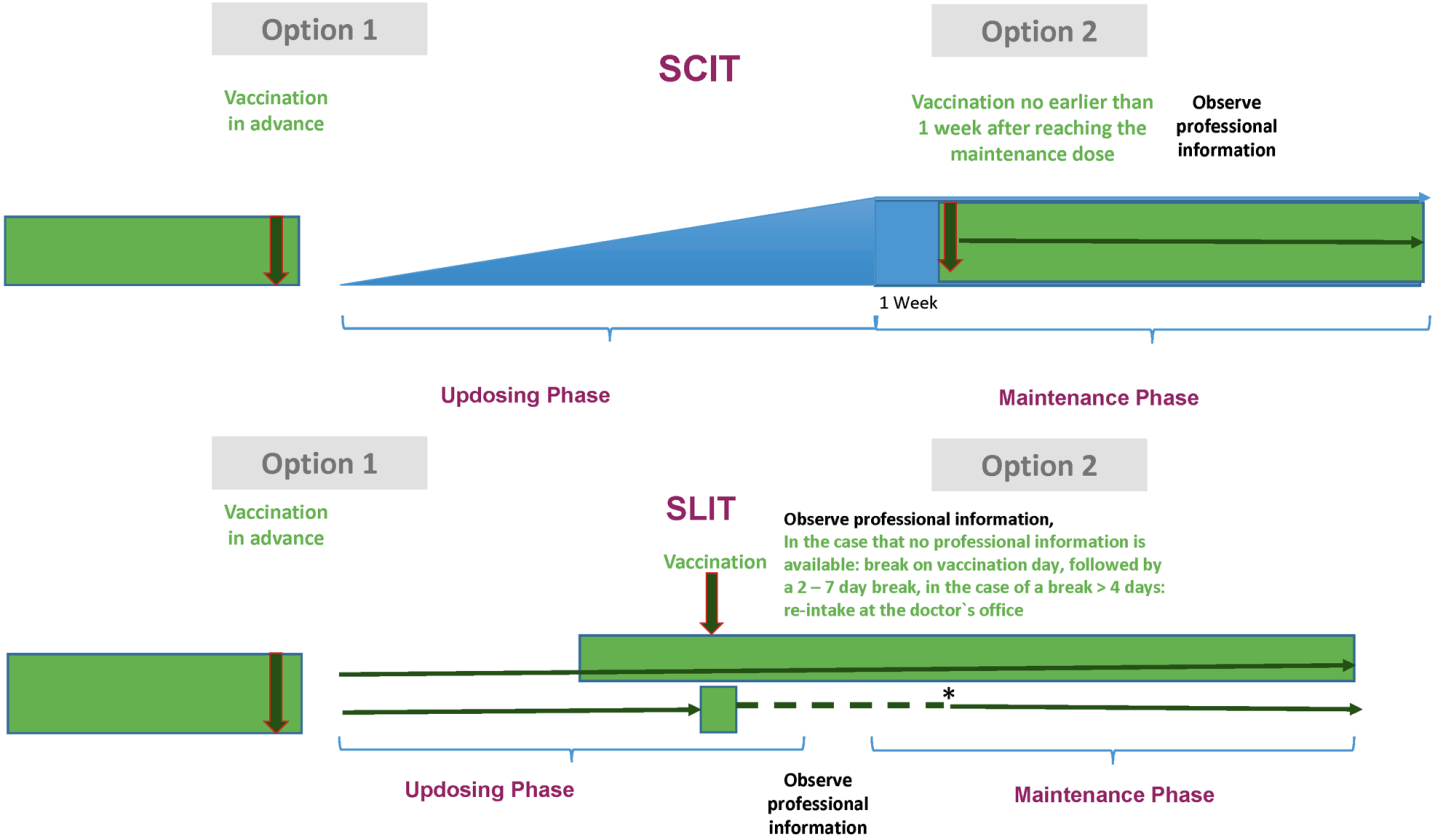
Recommendations for subcutaneous immunotherapy (SCIT) and sublingual immunotherapy SLIT. During the phases marked in green, vaccination can be carried out; during the phases marked in blue, there should be no vaccination.

The aim of this position paper is to present the use of AIT in its different forms of application (SCIT, SLIT, oral (OIT)) in the context of COVID-19 vaccination and to provide detailed recommendations for action (Figure 1). 

## Immune responses in AIT 

AIT attempts to induce a tolerogenic response to an individual antigen dose by continuous administration of that antigen. The main mechanisms include early desensitization of effector cells and progressive onset of a regulatory B and T cell response [34, 35, 36]. Although the main changes of specific immunotherapy are antigen-specific, recent data support a beneficial effect in restoring the general balance of the immune system altered toward Th2 immune response [37, 38]. A recent review analyzes the impact of the COVID-19 pandemic on the management of AIT in routine practice [39]. 

Because AIT is administered over a period of several years, there is considerable experience in coadministration with antiviral and other antimicrobial vaccines. Here, negative influences have not become known. On the contrary, immunologically, a restored balance of the innate immune system could improve its protective function [40]. 

## General information on COVID-19 vaccines 

COVID-19 has caused circa 110 million illnesses worldwide and claimed 2.5 million lives as of the writing of this review [41]. 

Effective vaccination against the novel virus represents an essential strategy to achieve maintenance of health care and public life while reducing social constraints [42]. 

Four vaccination goals exist in the context of COVID-19 vaccination. Most important is the prevention of severe COVID-19 courses and deaths. In addition, the protection of individuals at particularly high occupational risk of infection, prevention of disease transmission, and maintenance of public life are also important [43]. 

Due to extensive collaborations between academia and industry, new vaccine platforms were developed at an unprecedented pace less than a year after the discovery of the SARS-CoV-2 viral sequence, extensively and comprehensively tested in clinical trial programs, and approved (some provisionally or conditionally) after thorough review by regulatory authorities. 

## COVID-19 vaccines approved in Germany 

Worldwide, 66 COVID-19 vaccines are currently in clinical development and another 176 are in a preclinical development phase [44]. 

Vaccines against viral infectious diseases are designed to induce humoral and cellular immune responses against the vaccinated antigen. For this purpose, classical technologies are used, but also vaccine variants that have never been approved for human pharmacology [44]. 

The most common route of vaccine application is intramuscular injection, which induces strong priming of dendritic cells. However, vaccines using other routes of application are also currently being developed and their pharmacological properties and effects in terms of immune induction remain to be seen [45]. 

Traditionally, either complete viruses are used, or pathoimmunologically significant parts of the virus (for example, those necessary for entry into somatic cells). 

The mRNA-based SARS-CoV-2 vaccines from BioNTech/Pfizer and Moderna break new ground in terms of vaccine antigen delivery. BNT162b2 and mRNA-1273 are mRNA-based vaccines that do not introduce the antigen against which an immune response is to be induced (surface protein of the SARS-CoV-2 virus) but the blueprint (the mRNA) to produce the target protein in human cells. Primarily, the mRNA is taken up in muscle cells, but dendritic cells are critically involved in the presentation of the antigen. Regional lymph nodes are then where the immune response predominantly occurs. The target cells produce the viral antigen based on the information of the mRNA by transcription into the amino acid sequence of the protein structure, which becomes visible to the immune system of the vaccinee as a surface protein of such “transduced” cells. The immune system recognizes the surface protein of the SARS-CoV-2 as foreign and starts a humoral and cellular immune response. Degradation products of the produced proteins (peptides) are presented to the T cells, which are thus activated. This enables them to recognize and kill virus-infected cells. Furthermore, activated T cells support the antibody-producing B cells. 

Due to the rapid degradation of mRNA, it must be packaged in liposomes as a protective envelope so that uptake into the body’s own cells (the transfection) works. Their production has been improved in recent years, but liposomes and the mRNA they contain break down very easily, so these vaccines must be stored at very low temperatures (–20 °C (mRNA-1273) to –70 °C (BNT162b2) between production and use in humans. The lipid nanoparticles and their components also appear to be responsible for the severe allergic reactions to mRNA vaccines [32, 33]. 

Vector vaccines are another new class of vaccines. The AstraZeneca vaccine (ChAdOx1-S-(AZD1222)), the vaccine Ad26COV2.S from Janssen Pharmaceutical (Johnson & Johnson), and Sputnik V (Gam-COVID-Vac Adeno-based (rAd26-S+rAdS-S)) from the Moscow Gamaleja Institute are based on harmless human or monkey viruses that are unable to self-replicate and contain the SARS-CoV-2 surface protein [46]. 

The adenovirus-based vector vaccines can be stored in the refrigerator at 4 °C for several months without losing their efficacy. 

A disadvantage of virus vector vaccines in general is that they cannot be used to vaccinate several times in succession, because the vaccinee develops neutralizing antibodies against the vector itself. As a result, booster vaccinations can be only reduced or no longer effective. This is not a problem with Janssen Pharmaceutical’s Ad26COV2.S vaccine, which only needs to be administered once. When booster vaccination is necessary, an alternative strategy in cases of initial immunization with vector virus type A is booster using a heterologous vector type B vaccine. This has already become a reality in the randomized placebo-controlled trial with the Sputnik V vaccine developed in Russia recently [46]. 

## Immunology of type 1 allergies 

The type1 allergic reaction is based on a T2 immune response in which immunoglobulin E (IgE) and the cytokines interleukin (IL)-4, IL-5, and IL-13 in particular are significantly involved. From the use of biologics, we know that these key elements of T2 inflammation do not play a role in the antiviral immune response with respect to either IgE or the aforementioned cytokines. The immune response to anti-viral vaccination was not attenuated [47, 48], whereas anti-IgE treatment increased the production of type 1 interferon from dendritic cells, thereby enhancing the antiviral response [26, 49, 50]. 

## Practical recommendations for allergen immunotherapy 

### SCIT/SLIT/OIT in temporal connection with COVID-19 vaccinations 

In principle, manufacturer-specific guidelines Summary of Product Characteristics must be taken into account in the temporal connection of AIT and vaccinations. Thus, there should generally be an interval of ~ 1 week between SCIT and COVID-19 vaccination. Based on experience with other vaccinations, the following procedure has proven effective [25, 40, 51]. 

### Induction phase 

If it is possible to carry out the boosting phase of AIT completely before the planned vaccination date, this can be done as usual, and the recommendations given under “Maintenance therapy” then apply. If vaccination is imminent, initiation of SCIT, SLIT, or OIT should be delayed until 1 week after the second vaccination date [25]. 

### Maintenance therapy 

For continuation of ongoing AIT, we recommend a period of ~ 1 week between SCIT and vaccination, analogous to the above procedure, as well as at least 1 week interval after vaccination, observing the minimum interval between 2 SCIT applications recommended by the manufacturer. 

For SLIT or OIT, there are different recommendations from different manufacturers on the interval between vaccination and previous and subsequent SLIT or OIT administration. Therefore, no general recommendation can be given, but the information in the Summary of Product Characteristics should be considered and an individual decision should be made thereafter. In order to be able to limit possible side effects of the SLIT or OIT or the vaccination, we recommend to pause the SLIT or OIT on the day of the vaccination and to continue it with a certain time lag (2 – 7 days). In this case, SLIT or OIT can be applied up to the day before the vaccination [25]. 

Allergic reactions to vaccines are very rare, occurring at 1 per 1,000,000 – 30 per 100,000 vaccinations [52, 53, 54, 55, 56, 57]. 

To date, there is no evidence that they are increased under AIT [25, 40]. 

## Discussion and summary 

Currently, there is no scientifically substantiated evidence for clinically relevant interactions between AIT and the COVID-19 vaccines currently available in Germany. 

Both mRNA vaccines against SARS-CoV-2 are based on the same lipid-based nanoparticle carrier technology; the other two vaccines are vector vaccines. 

Additional vaccines are expected to be licensed in the coming months, and it is almost inevitable that adverse drug reactions will occur in the coming months that were not observed in the studies conducted for marketing authorization. Such real-life data will play a significant role in assessing interactions with other drugs – including AIT. 

Vaccine safety requires a proactive approach to maintain public confidence and reduce reluctance to vaccination among segments of the population. Vigilance, careful response, documentation, and characterization of these events are necessary to allow definition of mechanisms and appropriate approaches for prediction, prevention, and treatment. This is especially true for potential interactions of allergy therapies such as AIT. 

## Funding 

This work has been funded by German Allergy Societies AeDA and DGAKI. 

## Conflict of interest 

L. Klimek reports research funding, grants, and/or honoraria in the last 3 years from Allergopharma, Bioprojet, Biomay, Circassia, Viatris, HAL Allergie, ALK Abelló, Aimmune, Immunotek S.L., LETI Pharma, Stallergenes, Quintiles, Sanofi, ASIT Biotech, Lofarma, Thermofisher, Roxall, Allergy Therapeutics, AstraZeneca, GSK, Inmunotek, AeDA, Pohl Boskamp GmbH, Paul Martini Foundation, outside the submitted work; and is a member of the following organizations: AeDA, DGHNO, German Academy of Allergology and Clinical Immunology, German Allergy League; ENT-BV, GPA, EAACI. 

O. Pfaar reports grants and/or honoraria from ALK-Abelló, Allergopharma, Stallergenes Greer, HAL Allergy Holding B. V./HAL Allergie GmbH, Bencard Allergie GmbH/Allergy Therapeutics, Inmunotek S.L., Lofarma, Biomay, Circassia, ASIT Biotech Tools S. A., Laboratorios LETI/LETI Pharma, MEDA Pharma/MYLAN, Anergis S. A., Mobile Chamber Experts (a GA2LEN Partner), Indoor Biotechnologies, GlaxoSmithKline, Astellas Pharma Global, EUFOREA, ROXALL Medizin, Novartis, Sanofi Aventis, Sanofi Genzyme, Med Update Europe GmbH, streamedup! GmbH, Pohl-Boskamp GmbH, John Wiley and Sons AS, Paul Martini Foundation (PMS), Regeneron Pharmaceuticals Inc., Ingress-Health HWM during the last 36 months and all outside the present work. 

R. Brehler reports honoraria from ALK Abelló, Allergopharma, Allmiral, AstraZeneca, Bencard, Gesellschaft zur Förderung der Dermatologischen Forschung und Fortbildung e.V., GSK, HAL Allergie, LETI Pharma, MedUpdate, Merck, Novartis, Sanofi, Stallergenes, outside the submitted work; and membership in the following organizations: AeDA, DGAKI, EAACI, ABD. 

M. Wagenmann reports honoraria from ALK-Abello, Allergopharma, AstraZeneca, Bencard, Genzyme, GlaxoSmithKline, HAL Allergy, LETI, Meda Pharma, Novartis, Sanofi Aventis, Stallergenes, all outside the present work. 

M. Worm reports other/other conflicts of interest by Regeneron Pharmaceuticals, DBV Technologies S.A, Stallergenes GmbH, HAL Allergy GmbH, Bencard Allergie GmbH, Allergopharma GmbH & Co. KG, ALK-Abelló Arzneimittel GmbH, Mylan Germany GmbH, Leo Pharma GmbH, Sanofi-Aventis Deutschland GmbH, Aimmune Therapeutics UK Limited, Actelion Pharmaceuticals Deutschland GmbH, Novartis AG, Biotest AG, AbbVie Deutschland GmbH & Co. KG, Lilly Deutschland GmbH, all outside the scope of the present work. 

S. Becker reports honoraria from HAL Allergie GmbH, Bencard Allergie GmbH, Allergopharma GmbH & Co. KG, ALK-Abelló Arzneimittel GmbH, Mylan Germany GmbH, Sanofi-Aventis Deutschland GmbH, Novartis AG, AstraZeneca, Ambu, Karl Storz, outside the submitted work. 

N. Novak reports honoraria from Alk Abello, Stallergens Geer, Hal Allergy, Leti Pharma, Sanofi Genzyme, Abbvie, Leo Pharma, Novartis, streamed up, and Blueprint outside the submitted work. 

The other authors declare no conflicts of interest.
